# Efficacy and safety of Naoxintong capsule for acute ischemic stroke: A protocol for systematic review and meta-analysis

**DOI:** 10.1097/MD.0000000000027120

**Published:** 2021-08-27

**Authors:** Na Li, Fan Yang, Zhilong Zhao, Juan Jiang, Yi Lei

**Affiliations:** a Southwest Jiaotong University, Chengdu, China; b Institute of Laboratory Animal Sciences, Sichuan Academy of Medical Sciences & Sichuan Provincial People's Hospital, Chengdu, China; c Chengdu university of traditional Chinese medicine, Chengdu, China; d Sichuan Academy of Medical Sciences & Sichuan Provincial People's Hospital, Chengdu, China.

**Keywords:** acute ischemic stroke, meta-analysis, Naoxintong capsule, protocol

## Abstract

**Background:**

Acute ischemic stroke (AIS) is one of the most common causes of mortality and disability worldwide, which has become a global health concern due to the high prevalence, mortality, and disability rate. Naoxintong capsule is an oral Chinese patent preparation used extensively to treat AIS in China. However, the systematic evaluation on the clinical efficacy and safety of Naoxintong capsule is still absent. Therefore, we attempt to perform a systematic review and meta-analysis based on the existing evidence, in order to provide solid support for the clinical practice of Naoxintong capsule in the treatment of AIS.

**Methods:**

We will search both English and Chinese databases, including Scopus, EMBASE, PubMed, Cochrane library, Google Scholar, Web of Science, CNKI, VIP, Wanfang, and Chinese Biomedical Literature Database, for randomized controlled trials which focus on Naoxintong capsule treating AIS. The retrieving time was from inception to August 2021. According to eligibility criteria, 2 researchers will independently screen information and assess the quality of selected articles. The RevMan 5.3 (Copenhagen, The Nordic Cochrane Centre, The Cochrane Collaboration) software will be used for meta-analysis.

**Results:**

The clinical efficacy and safety of Naoxintong capsule in the treatment of AIS will be systematically evaluated or descriptive analyzed.

**Conclusion:**

The study will provide rigorous evidence to identify whether the application of Naoxintong capsule for treating AIS appeared to be adequate reliability regarding on the efficacy and safety.

**INPLASY registration number:**

INPLASY202180052

## 1 Introduction

Acute ischemic stroke (AIS), accounting for about 80% of all stroke cases is characterized by neurological dysfunction due to the focal occlusion or stenosis of arteries in the brain.^[1]^ Worldwide, AIS is the second leading cause of death and also the major cause of permanent disability.^[2]^ Moreover, the huge health-care costs consumed by AIS have imposed a heavy burden on society and family.^[3]^ Although trends in AIS incidence have apparent geographical variations, the high prevalence of AIS has led to an mounting demands on social-care systems around the world.^[4]^ As a consequence, AIS has become a global health concern. Currently, effective and specific treatments for AIS have not yet been accepted generally, except for thrombolytic therapy, which is aiming to restore cerebral blood flow.^[5,6]^ Nevertheless, the number of patients who are candidates for thrombolysis is still small, due to narrow therapeutic window. ^[7,8]^ In some cases, the use of thrombolytic treatment may result in symptomatic intracerebral hemorrhage, which has brought challenge to AIS management.^[9]^ Furthermore, the ischemia-reperfusion injury following the vascular recanalization often evokes additional complications.^[10]^ Therefore, it is crucial to explore novel therapeutic approach to improve efficacy and safety of treatment for AIS.

Brain injury caused by AIS develops a from a series of complicated pathophysiological events which involving oxidative stress, neuroinflammation, excitotoxity, neuronal apoptosis, and peri-infarct depolarizations.^[11]^ Modern researches have revealed that a large number of traditional Chinese medicine hold the effect of alleviating such pathophysiological changes above.^[12]^ Additionally, with the unique theoretical system of etiology, diagnosis, and treatment, traditional Chinese medicine have been used to treat AIS in China for a long history, and consequently, many empirical prescriptions have been developed for AIS therapy based on the extensive experience.^[12]^ Naoxintong capsule is derived from a classic prescription named Buyang Huanwu decoction, and a Chinese patent medicine approved by China Food and Drug Administration which has been used to treat AIS in China.^[13]^ In recent years, multiple clinical studies have shown the neuroprotective effects of Naoxintong capsule in AIS, which can not only improve the brain function and ameliorate activities of daily living dependency bust also reduce the serum inflammatory markers and normalize the peripheral hemodynamics.^[14–16]^ Biologically, the neuroprotective properties can be correlated to the actions of Naoxintong capsule on anti-oxidative stress, anti-inflammation, anti-apoptosis, etc.^[17,18]^

Overall, various clinical studies have suggested that Naoxintong capsule is beneficial in AIS. However, systematic evidence to support its efficacy and safety in the treatment of AIS remains unclear. Therefore, aiming to assess the quantity, quality and overall strength of existing randomized controlled trials (RCTs) evidence on Naoxintong capsule for AIS, we attempt to perform this systematic review and meta-analysis. We hope this study can help to identify whether the application of Naoxintong capsule appeared to be adequate reliability regarding on the efficacy and safety.

## 2 Methods

### 2.1 Registration

The current protocol of systematic review and meta-analysis, aiming to provide solid evidence for clinical practice of Naoxintong capsule in treating AIS, is prepared in accordance with the Preferred Reporting Items for Systematic reviews and Meta-Analyses for Protocols statement guideline. This protocol has been registered on INPLASY, with the unique registration number INPLASY202180052 (https://inplasy.com/inplasy-2021-8-0052/).

### 2.2 Eligibility criteria for included studies

#### 2.2.1 Type of studies

This study will employ RCTs which were published in English and Chinese to assess the efficacy and safety of Naoxintong capsule for AIS, regardless any limitation of blinding. Cohort studies, case reports, cross-over studies, and preclinical studies will be excluded.

#### 2.2.2 Type of participants

This study will encompass participants who were diagnosed as AIS by cranial computed tomography and/or magnetic resonance imaging, regardless of any age and gender. AIS patients with cerebral hemorrhage will be excluded.

#### 2.2.3 Type of interventions

Interventions can be Naoxintong capsule therapy used alone or in combination with other therapies, without limits on dose or duration of administration. Control interventions can be blank control, placebo control or other routine intervention control (eg, rt-PA, endovascular intervention, aspirin, and so on), while Naoxintong capsule treatment was not applied in control group.

#### 2.2.4 Type of outcomes

The major outcome in this study is neurological impairment degree, as measured by National Institutes of Health Stroke Scale and modified Rankin scale scores. The additional outcomes include activities of daily living dependency measured as Barthel Index score, total serious adverse events, and death.

### 2.3 Search methods for identification of studies

#### 2.3.1 Search strategy

We will search the Scopus, EMBASE, PubMed, Cochrane library, Google Scholar, Web of Science, CNKI, VIP, Wanfang, and Chinese Biomedical Literature Database from inception to August 2021 to retrieve relevant articles both in English and Chinese. Take PubMed as an example (Table 1), the key phrases include Naoxintong, AIS, humans, etc.

**Table 1:** Search strategy applied in PubMed.

Num	Entry terms
1	“Acute ischemic stroke” [MeSH Major Topic]
2	“Brain ischemia ” [MeSH Major Topic]
3	“Cerebral Infarction” [MeSH Major Topic]
4	“Cerebrovascular ischemia ” [Text word]
5	“Brain accident” [Text word]
6	“Infarction, Anterior Cerebral Artery” [Text word]
7	“Infarction, Middle Cerebral Artery” [Text word]
8	“Infarction, Posterior Cerebral Artery” [Text word]
9	“Apoplexy” [Text word]
10	#1 or #2 or #3 or #4 or #5 or #6 or #7 or #8 or #9
11	“Naoxintong” [Text word]
12	“Naoxintong capsule” [Text word]
13	“Buchangnaoxintong” [Text word]
14	#11 or #12 or #13
15	#10 and #14
16	Animals [MeSH Major Topic]
17	Humans [MeSH Major Topic]
18	#16 not #17
19	#15 not #18

#### 2.3.2 Searching other resource

Meanwhile, we will screen potential studies in the reference lists of valid studies as well as other relevant publications, as the information source for supplement. Moreover, unpublished data from clinical trial registries, and grey literature will be retrieved.

### 2.4 Data collection

#### 2.4.1 Study selection

**Figure 1:**
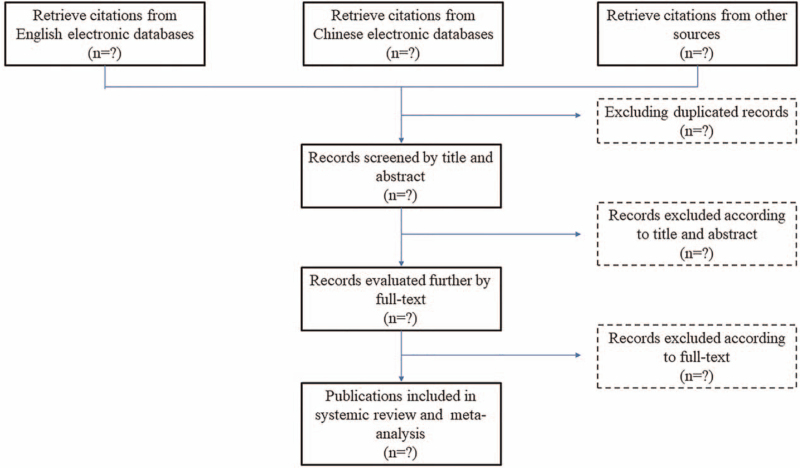
Flow chart of screening literature for meta-analysis.

According to the search strategy, relative studies will be retrieved and imported into Endnote software (Clarivate Analytics, Philadelphia, USA). After removal of duplication, all literature will be screened by titles and abstracts initially, and then followed by full-text. All procedures will be conducted by 2 authors independently. A third author will be invited to consult with to address disagreements, if there are any. Reasons for exclusion of studies will be documented and reported. The selection process will follow the flow diagram (Fig. 1) which in accordance with the Preferred Reporting Items for Systematic reviews and Meta-Analysis.

#### 2.4.2 Data extraction

Three initial articles will be used to pilot and refine to establish a standard extraction form, which contains the following domains: study information (eg, title, first author, language, magazine, year of publication, randomization, and blinding), participant information (eg, age, sex ratio, and sample size), intervention information (eg, type, course, frequency and dose of treatment in experimental, and control groups), and outcomes (indexes of major and minor outcomes). Available data will be extracted by 2 independent reviewers from full text, and disagreements will be resolved by consult with a third one. Numerical data will be extracted from tables, text or figures. If the data were not detailed, WebPlotDigitizer (PLOTCON, Oakland, CA) will be used to extract data from graph. The original authors of articles will be contacted through email in case of missing data. If no response, the incomplete article will be excluded.

### 2.5 Assessment of risk of bias

By using the Cochrane Collaboration Handbook,^[19]^ 2 independent authors will assess the methodological quality of the included studies through 7 domains (random sequence generation, allocation concealment, blinding of participants and personnel, blinding of outcome assessors, incomplete outcome data, selective reporting, and other bias). Each domain will be considered low, unclear or high risk of bias. A third author will be consulted with to settle disagreements in case of any variations in opinion.

### 2.6 Statistical analysis

The RevMan 5.3 software will be used for meta-analysis. The relative risk will be used as the effect index for the dichotomous variable, standardized mean differences will be used as the effect index for the continuous variable. The confidence interval of each effect index was set to 95%. I^2^ statistic will be adopted to assess the heterogeneity. If there is without heterogeneity between the studies (I^2^ ≤ 50%), the fixed effect model will be utilized; Otherwise (I^2^ > 50%), the random effects model will be selected. *P* < .05 indicates statistical significance. In addition, if the clinical data provided by the included literatures are incomplete and cannot be systematically evaluated, the descriptive analysis shall be carried out.

When there is high heterogeneity across included studies, subgroup analysis will be conducted according to the age, gender, treatment course, dosage of included patients, if adequate information could be available from include studies. We will also use the sensitivity analysis to assess the robustness of the findings, and low-quality literature will be excluded. If necessary, funnel plot and Egger test will be performed to quantitatively analyses the potential publication bias.

### 2.7 Grading quality of evidence

Two authors will independently appraise the strength of the evidence in compliance with Grades of Recommendation, Assessment, Development, and Evaluation guidelines. The definitions of quality in the Grades of Recommendation, Assessment, Development, and Evaluation system are high, moderate, low, and very low.^[20]^

### 2.8 Ethics and dissemination

Ethical approval is not required, because this study is based on existed literature. The findings of this systematic review will be disseminated through a peer-reviewed journal.

## 3 Discussion

AIS is the leading cause of death and long-term disability worldwide. Brain injury following the occurrence of AIS is a public health issue around the world.^[21]^ The general approach to the management of AIS is thrombolysis, however, the narrow therapeutic window, bleeding complications, and ischemia-reperfusion injury limit the application of thrombolytic treatment.^[22]^ Therefore, novel treatment approach is needed to improve AIS outcome. Naoxintong capsule is a traditional Chinese patent medicine that consists of 16 traditional Chinese medicines.^[23]^ Mounting RCTs have shown the efficacy and safety of Naoxintong capsule in the treatment of AIS, however, these findings are yet to conclusively confirmed. Systematic review and meta-analysis provide an available approach to assess the existing evidence to support the clinical practice. Therefore, in order to give rigorous evidence and scientific guide for the application of Naoxintong capsule treating AIS, the systematic and meta-analysis of reported RCTs will be performed strictly following the Cochrane Handbook for Systematic Reviews of Interventions.^[19]^

## Author contributions

**Conceptualization:** Na Li.

**Formal analysis:** Na Li, Zhilong Zhao.

**Funding acquisition:** Yi Lei.

**Methodology:** Fan Yang, Juan Jiang.

**Writing – original draft:** Na Li, Fan Yang.

**Writing – review & editing:** Yi Lei.
